# B7-H3 as a Target for CAR-T Cell Therapy in Skull Base Chordoma

**DOI:** 10.3389/fonc.2021.659662

**Published:** 2021-11-15

**Authors:** Cheng Long, Gaowei Li, Chengyun Zhang, Tao Jiang, Yanjun Li, Xin Duan, Gang Zhong

**Affiliations:** ^1^ Orthopedics Department, West China Hospital, Sichuan University, Chengdu, China; ^2^ Department of Neurosurgery, West China Hospital, Sichuan University, Chengdu, China; ^3^ Orthopedics Department, Xiandai Hospital of Sichuan Province, Chengdu, China; ^4^ Orthopedics Department, Fukang Hospital of Tibet, Chengdu, China

**Keywords:** B7-H3, chordoma, CAR-T cells, skull base chordoma, immunotherapy

## Abstract

**Objective:**

chordomas are rare bone tumors with few therapeutic options. Skull base and sacrum are the two most common origin sites. Immunotherapies are emerging as the most promising approaches to fight various cancers. This study tends to identify new cell surface targets for immunotherapeutic options of skull base chordomas.

**Methods:**

we profiled 45 skull base chordoma clinical samples by immunohistochemistry for the expression of six CAR-Targets (PD-L1, B7-H3, B7-H4, VISTA, HER2 and HER3). In addition, we generated B7-H3 targeted CAR-T-cells and evaluated their antitumor activities *in vitro*.

**Results:**

We found that B7-H3 was positively stained in 7 out of 45 (16%) chordoma samples and established an expression hierarchy for these antigens (B7-H3 > HER3 > PD-L1 > HER2 = VISTA = B7-H4). We then generated a B7-H3 targeted CAR vector and demonstrated that B7-H3-CAR-T-cells recognized antigen positive cells and exhibited significant antitumor effects, including suppression of tumor spheroid formation, CAR-T-cell activation and cytokine secretion.

**Conclusions:**

Our results support B7-H3 might serve as a promising target for CAR-T-cell therapies against chordomas.

## Introduction

Chordoma is a rare tumor arising from notochordal remnant, mainly involving the skull base, sacrococcygeal area and vertebral bodies (1). At present, chemotherapeutic options are limited and the principal treatment of chordoma is surgical resection and radiotherapy (1–3). Chordoma is often not completely resectable and shows a high recurrence rate and shortened survival, especially in skull base where complete tumor resection is often not possible because of the proximity of cranial nerves (4, 5). Therefore, there is a great need for efficient therapies that reduce recurrence and progression.

Chimeric antigen receptor (CAR) T cells are patient derived T cells that have been genetically engineered to express an engineered synthetic receptor for targeting and killing cancer cell (6). With the unprecedented success of CAR-T cells in blood cancer, a growing number of studies have focused on translating this treatment to solid tumors. In contrast to CAR-T cell therapy for hematological malignancies, CAR-T cell therapies for solid tumors have shown limited antitumor effects in early phase clinical trials (7). Some of the challenges for CAR-T cell therapy against solid cancers include a paucity of specific target antigens, limited trafficking to tumor sites, antigen heterogeneity and loss, as well as the immunosuppressive tumor microenvironment (8). However, in recent years many promising strategies have been reported to improve the antitumor effects of CAR-T cells in a variety of solid tumors, such as dual CAR designs that recognize multiple antigens at once (9), combining with oncolytic virus (10), expression of cytokines or chemokines (11), and depletion of other suppressive factors in the microenvironment (12).

Several CAR-T targets have been reported as promising targets in solid tumors, including HER2, HER3, Mesothelin, GPC3, CLDN18.2, and PSMA (13). So far, except for EGFR, few cell surface antigens were known to be overexpressed in chordoma (14, 15). Recently, several B7 family members, including B7-H3, B7-H4, VISTA and PD-L1, have been frequently reported to be overexpressed on various cancers, implicating they may serve as potential targets for CAR-T therapy (16–19). Unlike PD-L1, the receptors of B7-H3 and B7-H4 remain unknown. B7-H3, also known as CD276, is highly expressed in multiple tumor tissues and its expression is limited in normal tissues. This molecule has an immunosuppressive function by reducing the cytotoxic activity of type I interferon (IFN) released by T cells and natural killer cells (20). The mRNA of B7-H4 is widely expressed in various tumor and normal tissues, but in protein level, it is only detected in tumor tissues (21). B7-H4 has the function of regulating immune response by delivering costimulatory signals (22). Thus, a comprehensive comparison of the expression of these targets in chordoma is much needed.

In the present study, we profiled 45 skull base chordoma clinical samples by immunohistochemistry for the expression of 6 potential cell surface cancer immunotherapy targets (PD-L1, B7-H3, B7-H4, VISTA, HER2 and HER3). In addition, we generated B7-H3 targeted CAR-T-cells and tested their antitumor activities using patient derived tumor spheres.

## Materials and Methods

### Ethics

Patient samples for tumor antigen profiling were obtained under a West China Hospital approved protocol. Informed consent was obtained from all patients in accordance to the Declaration of Helsinki.

### Immunohistochemistry and Scoring System

Immunohistochemical (IHC) staining was performed by using the following primary antibodies: HER2 (#2242, Cell Signaling Technology), B7-H3 (#14058S, Cell Signaling Technology), PD-L1 (#13684, Cell Signaling Technology), HER3 (#12708, Cell Signaling Technology), VISTA (#54979, Cell Signaling Technology) and B7-H4 (#14572, Cell Signaling Technology), according to standard protocol as previously described []. The histoscore of the staining quantification was evaluated according to a previously described formula (strongly positive percent cells × 3) + (moderate positive percent cells × 2) + (weakly positive percent cells × 1), and the histoscore of pixel quantification was calculated as total intensity/total cell number (23).

### Flow Cytometry and Antibodies

The antibodies used to identify the phenotype of CAR-T cells included CD3-allophycocyanin (APC)-CY7 HIT3a, perforin-APC B-D48, GZMB-PE QA16A02, LAG3-FITC 11C3C65, TIM3-BV711 F38-2E2, and PD-1-BV605 NAT105 (all purchased from BioLegend). Mouse monoclonal antibody J42 was used to detect B7-H3 expression status on tumor cells (24).

### Cells Culture and Lentivirus Packaging

A375 wild type cell line and A375 cells with B7-H3 KO were gifts from Dr. Zongliang Zhang (24). Lentivirus packaging was performed using HEK293T cells transfected with target plasmid and two packaging plasmids psPAX2 and pMD.2G using polyethylenimine (Sigma). Forty-eight hours post transfection, the supernatants were collected, filtered with 0.45 μm filter, and further concentrated by ultracentrifugation at 100,000 g for 2 h. The concentrated lentivirus was resuspended in PBS and stored at −80°C until use.

### Vector Construction and CAR-T Cell Preparation

CAR was constructed with a lentiviral vector encoding a B7-H3-specific scFv (25), 4-1BB and CD3-ζ costimulatory domains, as well as a P2A sequence followed by CD19 with the cytoplasmic domain truncated (tCD19). Human peripheral blood mononuclear cells were isolated using density gradient centrifugation and cultured in X-vivo medium (Lonza) supplemented with antiCD3 (OKT3, 100 ng/mL; BioLegend), antiCD28 (CD28.2, 100 ng/mL; BioLegend), and IL-2 (100 units/mL; Life Science). After forty-eight hours, activated cells were transfected with lentivirus and cultured continuously for two weeks. Control T cells were transduced with tCD19 lentivirus and cultured under the same conditions.

### Tumorsphere Culture and Assay

Fresh surgical chordoma tissue was mechanically cut into approximately 1-2 mm^3^ pieces and incubated with collagenase (Cat. No. C6885, Sigma) for 3-6 hours, and then cell suspension was filtered through a nylon mesh, washed with PBS and seeded on collagen coated plates using Iscove/RPMI 4:1 (Life Technologies, Carlsbad, CA) supplemented with 20% of fetal bovine serum. Cells were passed when 80% confluence was reached. Tumor sphere was cultured in matrigel (BD Biosciences) coated ultra-low attachment plate (Corning, CLS3471) using the 3D tumorsphere medium from PromoCell (C-28070) according to the instructions. Passage the tumorsphere culture before they start to develop a dark center. About ten days after passage, chordoma tumor spheres were treated with CAR-T cells at a 2:1 E:T ratio. The cell number in tumor spheres can be estimated by counting the control wells after trypson digestion. The diameter of tumor spheres can be calculated by manual measurement under light microscope assisted with computer-based imaging software, and then the culture supernatants and cells were subjected to ELISA and FACS assays respectively. Human TNF alpha (ab181421) and IFN gamma (ab46025) were purchased from Abcam (Cambridge, MA).

### Statistics

Data are expressed as mean ± SEM. Unpaired 2-tailed Student’s t tests were performed by using GraphPad Prism 8. p < 0.05 was considered to be significant, and the significance levels are represented as ∗p < 0.05, ∗∗p < 0.01, and ∗∗∗p < 0.001.

## Results

### Expression Profiling of Six Tumor Antigens in Chordoma

**Figure 1:**
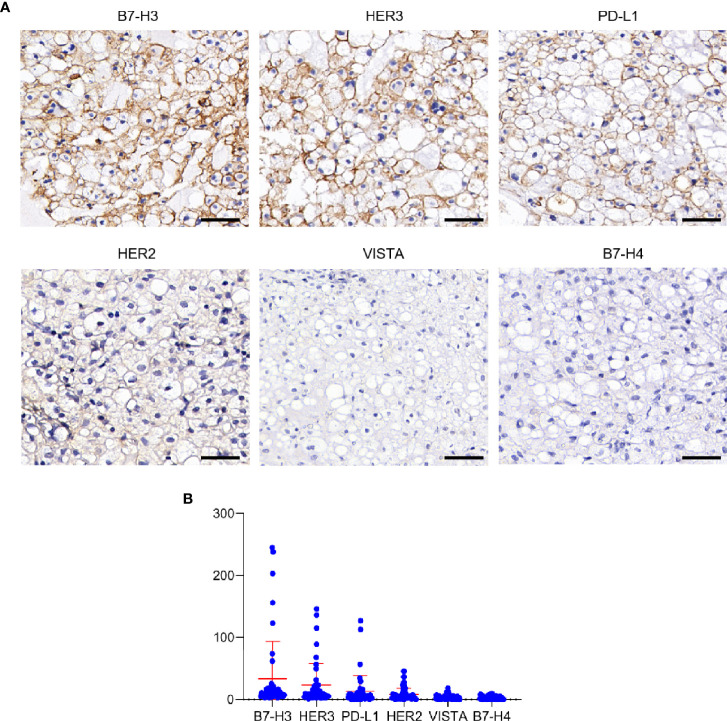
Expression analysis of target antigens in Chordoma. **(A)** Representative IHC staining images of different antigens, including B7-H3, HER3, PD-L1, HER2, VISTA and B7-H4. Scale bar: 50 μm. **(B)** Histoscore (H-score) of tumor sections (evaluated by pathologist blinded to tumor antigens and expression status).

To identify potential CAR-Targets for chordoma, we evaluated 6 tumor antigens (PD-L1, B7-H3, B7-H4, VISTA, HER2 and HER3) by IHC in a cohort of 45 skull base chordoma clinical samples (**Figure 1A**). Patient demographics and tumor features were listed in **Table S1**. As a result, seven (16%) were positive for B7-H3, with three of them demonstrating high–intensity staining (H-score > 200), two medium staining (H-score > 100) and two modest staining (H-score > 50), and the remaining 84% (38/45) show detectable but low expression. Six (13%) show modest to medium intensity staining for HER3. Three section samples were positively stained with PD-L1. We also observed low expression of HER2 in several cases. Most of these samples were negatively stained with VISTA and B7-H4 (**Figure 1B**).

### B7-H3-Retargeted CAR-T Cells Exhibited Cytolytic Activity Against Antigen Positive Tumor Cells

**Figure 2:**
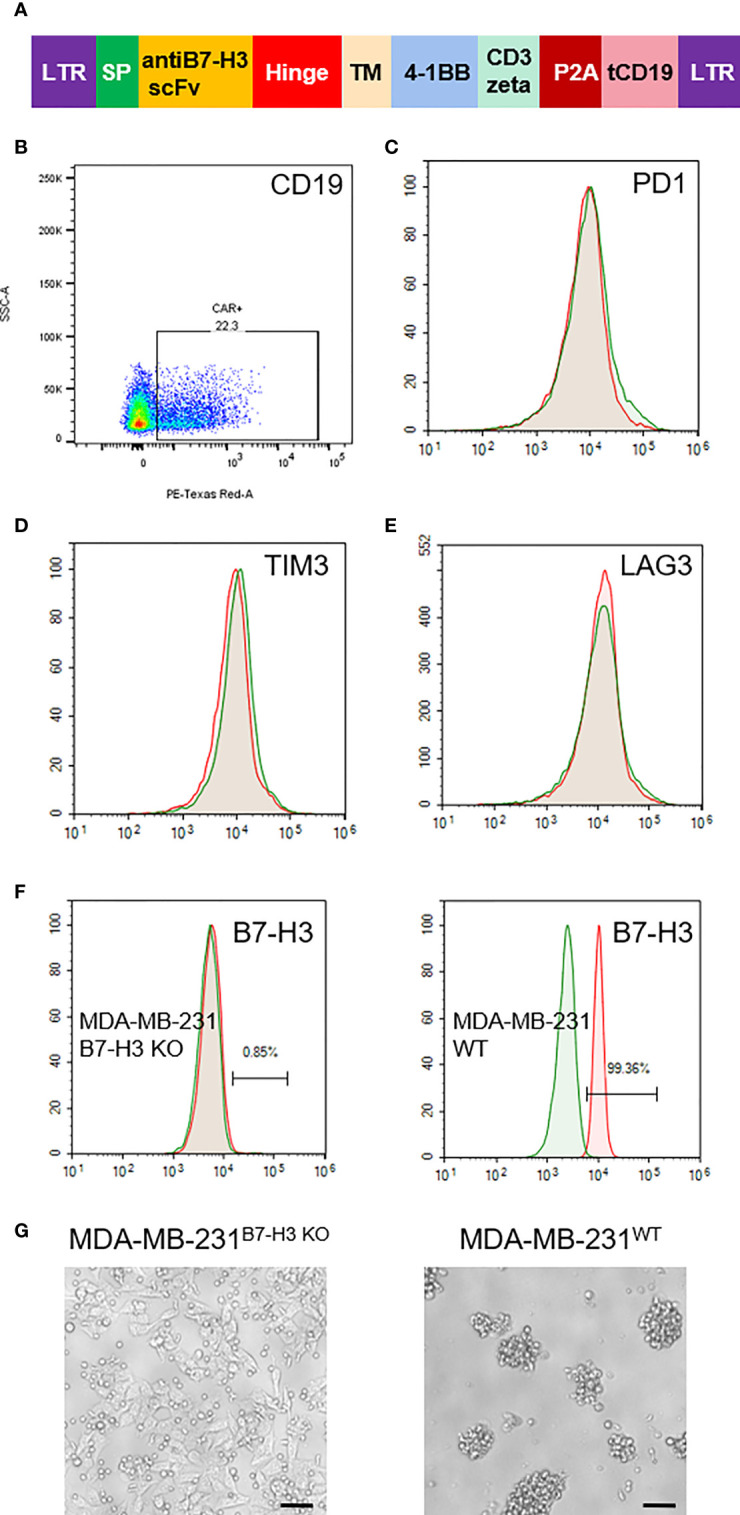
Generation of B7-H3-retargeted CAR-T cells. **(A)** Scheme of lentivirus vector; SP, signal peptide; TM, transmembrane domain; tCD19, truncated CD19. **(B)** FACS examination of the transduction efficiency of T cells by CAR lentivirus. **(C–E)**. FACS assays of the expression of PD-1, TIM3 and LAG3 on the CAR-T cells. **(F)** Expression examination of B7-H3 on MDA-MB-231 cells with B7-H3 KO and MDA-MB-231 wild type cells. **(G)** Cytotoxicity of B7-H3-retargeted CAR-T cells against MDA-MB-231^B7-H3 KO^ and MDA-MB-231^WT^ cells at a 2:1 E:T ratio. Scale bars: 50 μm.

Based on our IHC staining results, we constructed a lentivirus vector expressing B7-H3 re-directed CAR harboring a truncated CD19 as a marker for detection or tracking (**Figure 2A**). As shown in **Figure 2B**, the average transduction efficiency of T cells by this CAR expressing lentivirus was approximately 22%. Following transfection with lentivirus vector, we also examined the alteration of three T cell exhaustion markers by FACS. As shown, no significant changes were observed with the expression of PD-1, TIM3 and LAG3 on theCAR-T cells (**Figures 2C–E**). We further tested the specificity of theCAR-T cells with B7-H3 positive and KO cell lines (**Figure 2F**). As a result, B7-H3-retargeted CAR-T cells can recognize and kill MDA-MB-231^WT^ cells, but without effects on MDA-MB-231^B7-H3 KO^ cells (**Figure 2G**).

### B7-H3-Retargeted CAR-T Cells Suppressed Chordoma Tumor Sphere Formation

**Figure 3:**
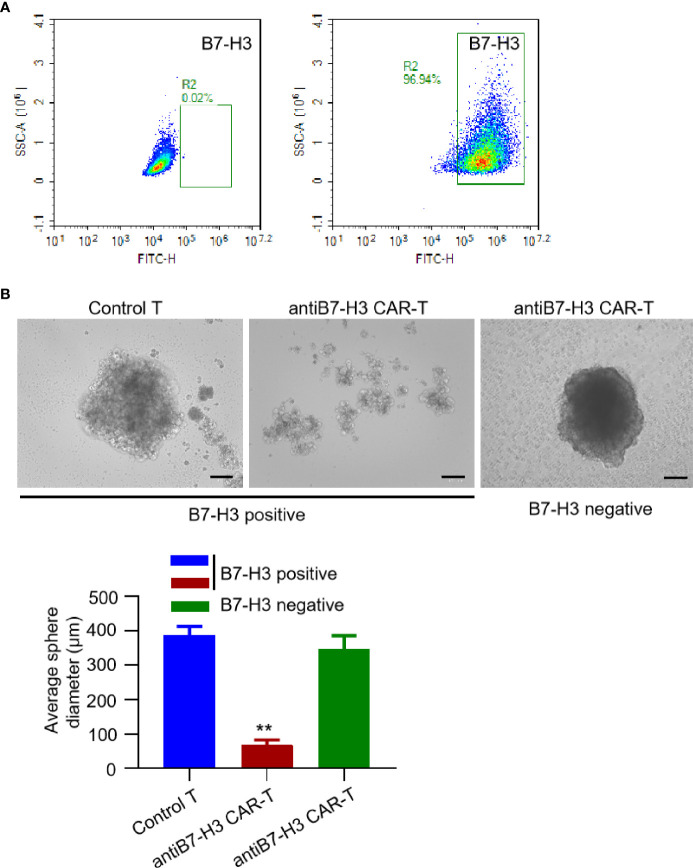
B7-H3-retargetedCAR-T cells suppressed chordoma tumor sphere formation. **(A)** Representative images of FACS examination of B7-H3 expression on primary chordoma cells isolated from two different clinical samples. **(B)** Representative images of B7-H3-retargeted CAR-T cells inhibited the tumor sphere formation of chordoma. Scale bars: 100 μm. **p < 0.01.

So far, there is no successful report of establish xenograft *in vivo* mouse model for chordoma. We also tried many times using NOG mice, but failed. However, we found that it is relatively easy to establish and propagate chordoma tumor spheres *in vitro*. Thus, we examined the activity of B7-H3-retargeted CAR-T cells by counting tumor sphere formation. Firstly, we screened the B7-H3 expression on the isolated primary chordoma cells by FACS. As shown in **Figure 3A**, tumor cells isolated from most of the chordoma samples were negatively stained (left panel), while several samples show high expression of B7-H3 (right panel) and were subjected to tumor sphere formation assay. From **Figure 3B**, it can be seen that B7-H3-retargeted CAR-T cells significantly inhibited the tumor sphere formation of chordoma (p < 0.01).

**Figure 4:**
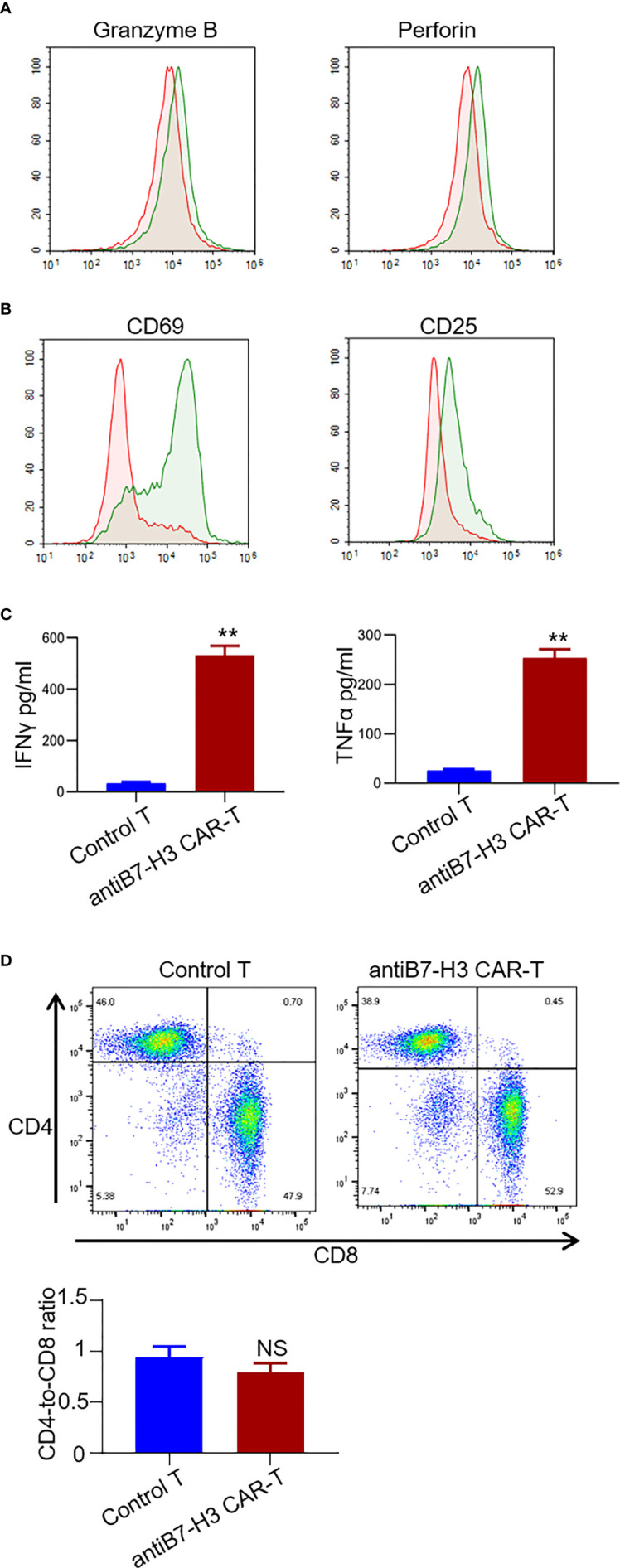
Characterization of B7-H3-retargetedCAR-T cells after co-cultured with chordoma cells. Forty-eight hours after co-culture of B7-H3-retargeted CAR-T cells with B7-H3 positive chordoma cells at a 2:1 E:T ratio, cells were subjected to FACS examination. **(A)** Secretion of Granzyme B and perforin from CD3 positive T cells was detected by FACS assay. **(B)** Up-regulation of activation markers CD69 and CD25 in CD3 positive T cells. **(C)** Concentrations of IFNγ and TNFα in the supernatants were measured by ELISA. **p < 0.01. **(D)** The phenotype analysis of CAR-T cells. 14 days after lentivirus CAR transduction, T cells were collected and stained with CD4-PE QA18A35 (Biolegend) and CD8-FITC SK1 (Biolegend), followed by FACS analysis. NS, not significant.

Next, we examined the secretion of granzyme B and perforin from CD3 positive T cells by FACS. An obvious increase was observed in the secretion of both granzyme B and perforin in B7-H3-retargeted CAR-T cells forty-eight hours after co-culture with chordoma cells at a 2:1 E:T ratio (**Figure 4A**). We also found a significant up-regulation of activation markers CD69 and CD25 in CD3 positive T cells (**Figure 4B**), as well as an augmentation in the secretion of IFNγ and TNFα in the supernatants after co-culture (p < 0.01.) (**Figure 4C**). The phenotype analysis showed that the CAR-T cells were uniformly positive for CD4 and CD8 with a ratio of 1:1.3 (**Figure 4D**).

## Discussion

Chordoma is a primary bone cancer with no approved therapy. Chordoma is resistant to standard chemotherapies, and despite surgical and/or radiation therapy, it often shows a high incidence of recurrence and progression with shortened survival and impaired quality of life (1–3). There is no known prognostic biomarker or therapeutic drug targets and the molecular mechanisms underlying chordoma development remain largely unexplored. The identification of therapeutic targets in this disease has been challenging mainly due to the rare occurrence of clinically actionable somatic mutations in chordoma (23, 26, 27). It was reported that the expression of PDGFR-α, EGFR and c-MET were increased in spinal chordoma (14, 15, 28), and tyrosine kinase inhibitor exhibited suppression effects on chordoma cells (29, 30). However, the overall clinical outcome of therapy with tyrosine kinase inhibitor is far from being satisfactory. Recent years, immunotherapy has emerged as one of the most promising therapeutic approaches against cancer (31). The aim of the present study was to identify new potential immunotherapy drug targets for chordoma.

For CAR-T therapy, the most important thing is to find a good tumor antigen as the target. The ideal therapeutic antigen target has a homogeneous and specific overexpression in tumor (13). In this study, we performed a comprehensive analysis of the expression status of six potential immunotherapy drug targets by IHC. As a result, B7-H3 was found to be homogeneously overexpressed in a small percentage of chordoma samples. Recent years, B7-H3 has emerged as an attractive immunotherapy target, because it is highly expressed across various tumor types with restricted expression in normal tissues (24, 32). Several B7-H3 targeted antibodies have entered into phase I/II clinical trials against multiple solid tumor types, such as enoblituzumab, an Fc-engineered antibody developed by MacroGenics, as well as antibody conjugated drug MGC018 and DS-7300a developed by MacroGenics and Daiichi Sankyo. The antitumor effects of B7-H3 targeted CAR-T cells have also been frequently reported both in preclinical and in clinical trials for glioma (33), anaplastic meningioma (34), pediatric brain tumors (35), as well as lymphoma (36). We also observed significant antitumor effects when co-cultured chordoma tumor spheres with B7-H3-targeted CAR-T cells. Our results support active preclinical and clinical exploration of B7-H3-targeted therapies for chordoma.

We found HER3 has a homogeneous and intermediate level expression in several chordoma sections. Genomic copy-number duplication of HER3 (ERBB3) has been reported in chordoma recently (27). Some FDA-approved tyrosine kinase inhibitors possess strong HER3 inhibition activity, such as afatinib and gefitinib. Furthermore, EGFR activation has also been reported, and an European multi-center trial is evaluating the efficacy of afatinib, an ERBB family (EGFR/ERBB1, HER2/ERBB2, ERBB3, and ERBB4) inhibitor, as first-line or later-line treatment in advanced chordoma (NCT03083678). Our findings provide evidence for exploring the efficacy of ERBB family inhibitors in the treatment of patients with chordoma.

It is notable that five samples were positively stained with PD-L1, and three out of the five samples show intermediate expression level. In recent years, the immune checkpoint blockers targeting PD-1/PD-L1 have achieved tremendous therapeutic success in various cancers (37, 38). These data support the rational usage of PD-1/PD-L1 blockers in clinical trials for chordoma therapy. HER2 is known drug target, and except breast it was highly expressed by various cancers. VISTA and B7-H4 were highly expressed in some cancers and were suggested to be promising targets for immunotherapy (17–19, 39). However, in this study, we failed to detect obvious expression of these three markers in chordoma.

In summary, we analyzed the expression status of six cell surface tumor antigen in a cohort of 45 chordoma samples. Our results indicated that B7-H3 is the most homogeneous and overexpressed antigen in chordomas, and B7-H3 targeted CAR-T cells significantly suppressed the formation of chordoma tumor sphere. We also detected low or intermediate expression level of HER3 and PD-L1 in chordomas. Given the low positive frequency of these targets, personalized therapy with careful selection of the patients, based on the expression status of the specific tumor antigens or markers, is anticipated in chordoma.

## Data Availability Statement

The original contributions presented in the study are included in the article/**Supplementary Material**. Further inquiries can be directed to the corresponding authors.

## Ethics Statement

The studies involving human participants were reviewed and approved by Ethics Board at Sichuan University. The patients/participants provided their written informed consent to participate in this study.

## Author Contributions

CL and GZ designed the experiments. CL, GL, and CZ collected the clinical samples, performed the IHC staining and data analysis. TJ, GL, and YL constructed the CAR vector and evaluated the CAR-T cell activities. CL wrote the paper. All authors contributed to the article and approved the submitted version.

## Funding

This study was supported by Major Subject of the Science and Technology Department of Sichuan Province (2017SZ0127 & 2019YFS0330) from China.

## Conflict of Interest

The authors declare that the research was conducted in the absence of any commercial or financial relationships that could be construed as a potential conflict of interest.

## Publisher’s Note

All claims expressed in this article are solely those of the authors and do not necessarily represent those of their affiliated organizations, or those of the publisher, the editors and the reviewers. Any product that may be evaluated in this article, or claim that may be made by its manufacturer, is not guaranteed or endorsed by the publisher.
