# Supplementary material for: B7-H3 as a Target for CAR-T Cell Therapy in Skull Base Chordoma
Source: Front Oncol. 2021 Nov 15;11:659662. doi: 10.3389/fonc.2021.659662 (PMC8634710; doi:10.3389/fonc.2021.659662)
Supplement: Supplementary file 1 [file Table_1.doc]

**Table S1. Patient Demographics and Tumor Features.**

| **ID** | **Age** | **Sex** | **Location** | **Pathology** |
| --- | --- | --- | --- | --- |
| **CHD1** | 46 | F | Clivus | Classic |
| **CHD2** | 58 | M | Clivus | Classic |
| **CHD3** | 55 | F | Clivus | Chondroid |
| **CHD4** | 37 | M | Clivus | Classic |
| **CHD5** | 63 | F | Clivus | Classic |
| **CHD6** | 29 | M | Clivus | Classic |
| **CHD7** | 13 | F | Clivus | Classic |
| **CHD8** | 35 | F | Clivus | Classic |
| **CHD9** | 28 | F | Clivus | Classic |
| **CHD10** | 47 | M | Clivus | Classic |
| **CHD11** | 42 | F | Clivus | Classic |
| **CHD12** | 34 | M | Clivus | Classic |
| **CHD13** | 65 | F | Clivus | Classic |
| **CHD14** | 31 | M | Clivus | Chondroid |
| **CHD15** | 23 | F | Clivus | Classic |
| **CHD16** | 17 | M | Clivus | Classic |
| **CHD17** | 29 | F | Clivus | Chondroid |
| **CHD18** | 48 | F | Clivus | Classic |
| **CHD19** | 56 | M | Clivus | Classic |
| **CHD20** | 39 | F | Clivus | Classic |
| **CHD21** | 42 | F | Clivus | Classic |
| **CHD22** | 44 | M | Clivus | Classic |
| **CHD23** | 22 | M | Clivus | Classic |
| **CHD24** | 33 | F | Clivus | Classic |
| **CHD25** | 31 | F | Clivus | Classic |
| **CHD26** | 50 | F | Clivus | Classic |
| **CHD27** | 68 | M | Clivus | Classic |
| **CHD28** | 42 | F | Clivus | Chondroid |
| **CHD29** | 26 | M | Clivus | Classic |
| **CHD30** | 58 | F | Clivus | Classic |
| **CHD31** | 16 | M | Clivus | Classic |
| **CHD32** | 42 | F | Clivus | Classic |
| **CHD33** | 31 | F | Clivus | Classic |
| **CHD34** | 67 | M | Clivus | Classic |
| **CHD35** | 26 | M | Clivus | Chondroid |
| **CHD36** | 37 | F | Clivus | Classic |
| **CHD37** | 53 | F | Clivus | Classic |
| **CHD38** | 36 | F | Clivus | Classic |
| **CHD39** | 61 | M | Clivus | Classic |
| **CHD40** | 42 | F | Clivus | Classic |
| **CHD41** | 52 | F | Clivus | Classic |
| **CHD42** | 35 | M | Clivus | Classic |
| **CHD43** | 46 | M | Clivus | Classic |
| **CHD44** | 29 | F | Clivus | Chondroid |
| **CHD45** | 55 | F | Clivus | Classic |
